# De Novo Analysis of the Transcriptome of *Meloidogyne* *enterolobii* to Uncover Potential Target Genes for Biological Control

**DOI:** 10.3390/ijms17091442

**Published:** 2016-09-01

**Authors:** Xiangyang Li, Dan Yang, Junhai Niu, Jianlong Zhao, Heng Jian

**Affiliations:** 1 Department of Plant Pathology, China Agricultural University, Beijing 100193, China; 20047801@bua.edu.cn (X.L.); yd13_0@163.com (D.Y.); jianlongzhao@cau.edu.cn (J.Z.); 2 Beijing University of Agriculture, Beijing 102206, China; 3 Tropical Crops Genetic Resources Institute, Chinese Academy of Tropical Agricultural Sciences, Danzhou 571737, China; niujunhai@tom.com; 4 Hainan Engineering Technology Research Center for Tropical Ornamental Plant Germplasm Innovation and Utilization, Danzhou 571737, China

**Keywords:** *Meloidogyne enterolobii*, nematode, transcriptome, effector, pathogenicity

## Abstract

*Meloidogyne enterolobii* is one of the obligate biotrophic root-knot nematodes that has the ability to reproduce on many economically-important crops. We carried out de novo sequencing of the transcriptome of *M. enterolobii* using Roche GS FLX and obtained 408,663 good quality reads that were assembled into 8193 contigs and 31,860 singletons. We compared the transcripts in different nematodes that were potential targets for biological control. These included the transcripts that putatively coded for CAZymes, kinases, neuropeptide genes and secretory proteins and those that were involved in the RNAi pathway and immune signaling. Typically, 75 non-membrane secretory proteins with signal peptides secreted from esophageal gland cells were identified as putative effectors, three of which were preliminarily examined using a PVX (pGR107)-based high-throughput transient plant expression system in *Nicotiana benthamiana* (*N. benthamiana*). Results showed that these candidate proteins suppressed the programmed cell death (PCD) triggered by the pro-apoptosis protein BAX, and one protein also caused necrosis, suggesting that they might suppress plant immune responses to promote pathogenicity. In conclusion, the current study provides comprehensive insight into the transcriptome of *M. enterolobii* for the first time and lays a foundation for further investigation and biological control strategies.

## 1. Introduction

Root-knot nematodes include over eighty species. These nematodes can negatively affect host plants in three main ways: damaging the plant roots, competing for nutrients and causing infection from other microbes induced by root damage [1]. *Meloidogyne* *enterolobii* (*M. enterolobii*) is an important obligate biotrophic plant parasite, infestation of which can lead to yellowing and stunting as a result of the nutritional and water stress caused by the physical root damage of nematode feeding [2]. *M. enterolobii* was first discovered in Danzhou, China, in 1983 [3] and has been spread and established in several continents and countries. *M. enterolobii* can infect a variety of host plants, including economic crops, such as cotton, pepper, tobacco and watermelon [2,4], and has become a potential threat to the agricultural economy in tropical and subtropical areas.

*M. enterolobii* has received great attention because of its ability to reproduce on a variety of host cultivars bred for nematode resistance. A PCR-based detection method can be used to identify *M. enterolobii* accurately [5]. Currently, there is no effective way of controlling *M. enterolobii*. Many efficient chemical-based nematicides have been banned or restricted for environmental and health concerns [6,7]. Biological control is considered an ideal alternative to the chemical methods, such as RNA interference (RNAi). However, detailed knowledge of the suitable target genes of *M. enterolobii* for such control strategies is still absent [8].

The interaction between plants and parasites is complicated and delicate. Plant parasitic nematodes have evolved to possess a variety of strategies that allow them to successfully infect and survive their hosts. For example, plant-parasitic nematodes can secrete numerous proteins, termed effectors, into their hosts that have various functions, including suppression of plant defenses and interference with plant signaling or hormone pathways to promote the formation of nematode feeding sites [9]. RNA sequencing is a high-throughput technique that can help identify the putative effectors and genes of the nematodes that are essential for their survival. To date, several nematodes have been successfully sequenced, including *Pratylenchus coffeae* [10], *Meloidogyne graminicola* [11], *Pratylenchus thornei* [12], *Heterodera avenae* [13] and *Aphelenchoides besseyi* [14], whereas transcriptome information of the more detrimental *M. enterolobii* is lacking. Therefore, the objective of the current research was to systematically study the transcriptome of *M. enterolobii* and to determine the possible mechanism of pathology of *M. enterolobii* in plants, as well as any important genes that might be used as RNAi targets for biological control. To achieve this objective, we collected the *M. enterolobii*, sequenced its transcriptome and carried out a series of bioinformatics analyses by comparing its transcriptome with those with known transcriptomic information.

## 2. Results

### 2.1. Assembly

**Figure 1:**
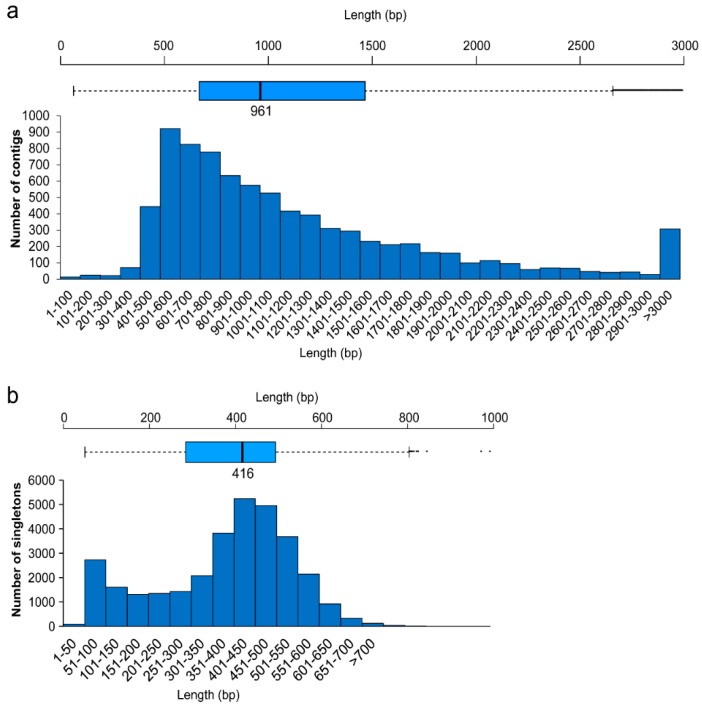
Distribution of *M. enterolobii* assembled reads. (**a**) Distribution of contigs of *M. enterolobii* contigs in different size ranges; (**b**) distribution of singletons of *M. enterolobii* in different size ranges.

Transcriptome sequencing of *M. enterolobii* generated 408,663 reads, for a total of 165,040,879 base pairs, with an average length of 403 bp, of which 355,760 reads were assembled into 8193 contigs with an average length of 1202 bp. Additionally, 31,860 singletons were obtained, with an average length of 380 bp. We obtained 8143 putative operons from the contigs and 29,403 from the singletons (Table 1). The lengths of most of the contigs and singletons were within the range of 500–600 bp. A detailed distribution is plotted in Figure 1.

### 2.2. GO Annotation

**Figure 2:**
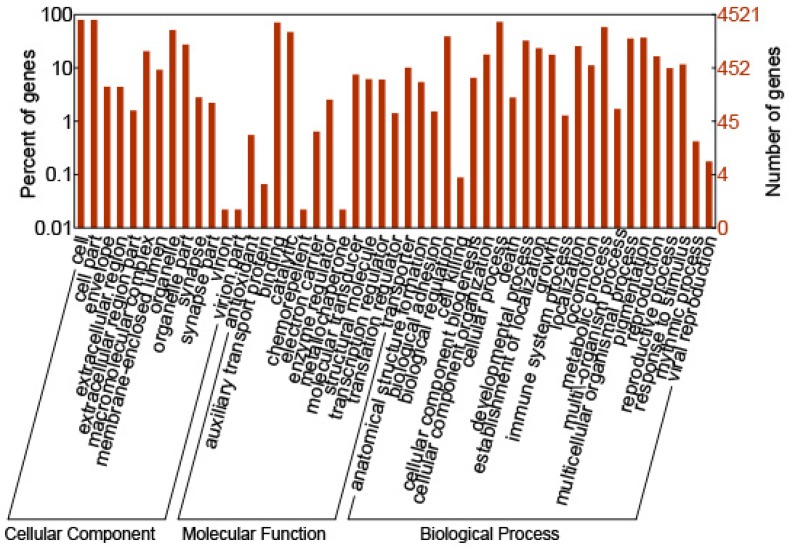
Functional classification of *M. enterolobii* transcripts. GO-annotated proteins of *M. enterolobii* were classified and plotted by WEGO according to the Gene Ontology Consortium.

Only 4521 of the 37,186 putative operons were successfully annotated. The GO annotations were mainly distributed into fifty-three functional groups that are involved in molecular function, cell components and biochemical processes. The annotated operons were derived from 2082 contigs and 2439 singletons, which accounted for approximately 12% of the total. Only nine GO functional groups contained over 30% of the total transcripts. Namely, they were cell part (71%), cellular metabolic process (52%), membrane-bounded organelle (44%), ion binding (44%), primary metabolic process (41%), organic substance metabolic process (41%), single-organism cellular process (41%), nitrogen compound metabolic process (39%) and biosynthetic process (31%; Figure 2).

**Figure 3:**
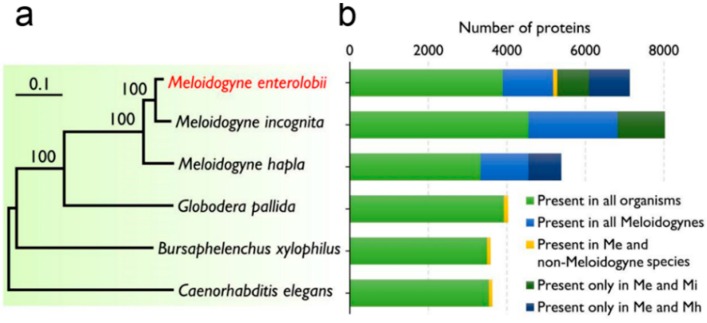
Phylogeny and ortholog distribution of six nematodes. (**a**) A genome-based phylogenetic tree generated with the neighbor-joining method; (**b**) The orthologous proteins identified in the species are represented using colored bars that are divided into five categories: present in all organisms (green), present in all *Meloidogyne species* (blue), present in *M. enterolobii* and non-*Meloidogyne* species (orange), present only in *M. enterolobii* and *M. incognita* (dark green) and present only in *M. enterolobii* and *M. hapla* (dark blue). Me: *M. enterolobii*; Mi: *M. incognita*; Mh: *M. hapla*.

The ortholog analysis was carried out by comparing *M. enterolobii* with several nematodes that have available genetic information, including *M. incognita*, *M. hapla*, *B. xylophilus*, *G. pallida* and *C. elegans*. Of the genes pooled from these nematodes, we identified 17,966 orthologous families and 42,156 non-orthologous families. Approximately 51% of the operons (18,977) from *M. enterolobii* belonged to 11,926 orthologous families, of which 2826 gene families, including 6999 operons, were unique to *M. enterolobii*. Phylogenetic analysis of 1022 conserved single-copy orthologous families showed that *M. enterolobii* had the closest relationship with *M. incognita*, followed by *M. hapla* (Figure 3a). We subdivided these orthologous families into five groups (Figure 3b). We further analyzed the gene families that were not shared by all of these nematodes. The results showed that nine of the functional GO subgroups existed in the three *Meloidogyne* species; two GO subgroups existed in *M. enterolobii* and non-*Meloidogyne* species; one GO functional subgroup existed in both *M. incognita* and *M. enterolobii*; and eleven were in both *M. hapla* and *M. enterolobii* (Table S1). KEGG pathway analyses showed that eight pathways were unique to *M. enterolobii*, including tetracycline biosynthesis (KO_00253; KO, KEGG Ontology), β-lactam resistance (KO_00312), bisphenol degradation (KO_00363), peptidoglycan biosynthesis (KO_00550), atrazine degradation (KO_00791), steroid degradation (KO_00984), biosynthesis of the siderophore group nonribosomal peptides (KO_01053) and malaria (KO_05144) (Supplementary Materials Table S2). The number of pathways that were unique to *M. incognita* and *M. hapla* were five and one, respectively. Compared with the other nematodes, three pathways had the highest KOs in *M. enterolobii*, including nicotinate and nicotinamide metabolism, carbon fixation pathways in prokaryotes and glycerolipid metabolism. Forty-one pathways were missing, but existed in the other two nematodes (Table S2).

### 2.3. Comparative Analysis of the Transcriptome of M. enterolobii

#### 2.3.1. CAZymes

**Figure 4:**
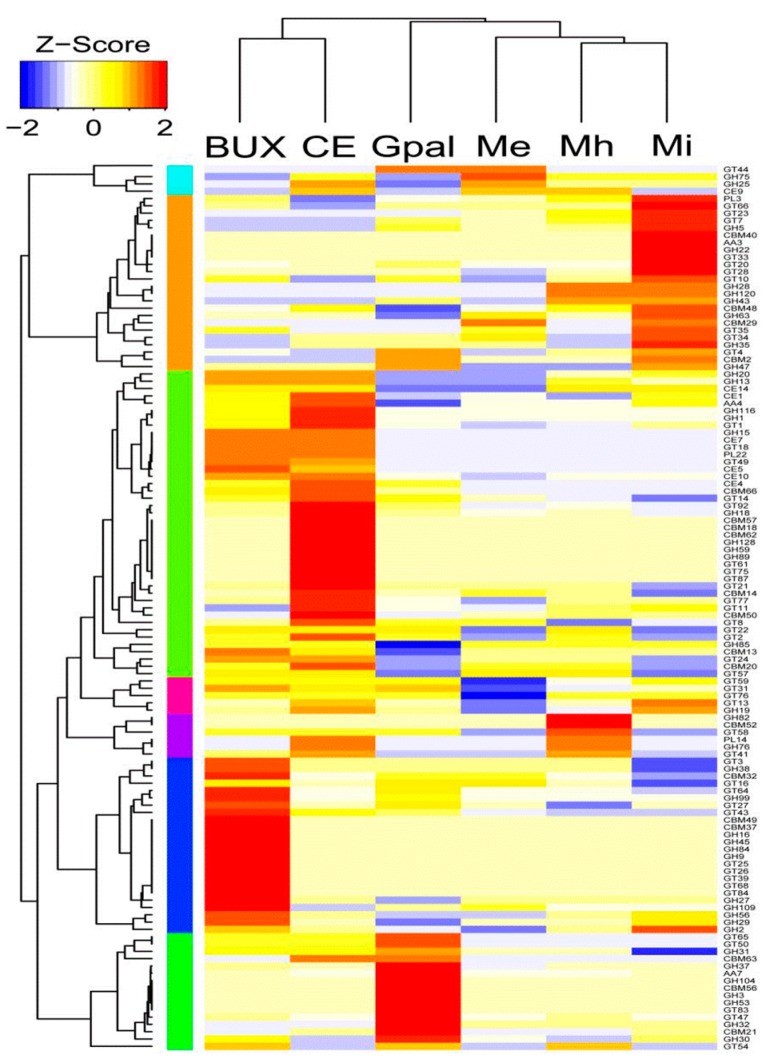
Comparison of the CAZyme repertoires in *M. enterolobii* and other selected nematode genomes. Enzyme families are represented by their family number according to the carbohydrate-active enzyme database (Available online: http://www.cazy.org/). Over-represented (yellow to red) and under-represented domains (yellow to blue) are depicted as Z-scores for each family. The figure was drawn using the R package heatmap.2. BUX: *B. xylophilus*; CE: *C. elegans*; Gpal: *G. pallida*; Me: *M. enterolobii*; Mi: *M. incognita*; Mh: *M. hapla*.

Searching CAZy and the dbCAN database for carbohydrate active enzymes (CAZymes), we found that *M. enterolobii* had only 249 CAZyme modules, which was the lowest among those of the six nematodes analyzed (Table S3). Typically, in the CAZyme modules, i.e., auxiliary activities, carbohydrate binding modules, carbohydrate esterase, glycoside hydrolase, glycosyltransferase and polysaccharide lyase, *M. enterolobii* had gene numbers to code for putative CAZymes as 2, 9, 3, 19, 31 and 1, respectively (Figure 4).

#### 2.3.2. Kinases

We identified kinases from six nematodes using Kinannote and compared the differences between these nematodes. The results showed that *M. enterolobii* had protein kinases in all twelve types of kinases. Compared with other nematodes, *M. enterolobii* had fewer kinases in most groups of kinases; for example, *M. enterolobii* had only one “RGC” kinase and two “atypical” kinases, which indicated the low abundance of these enzymes in *M. enterolobii* (Table S4).

#### 2.3.3. Neuropeptides

Examining the *flp* and *nlp* coded neuropeptides showed that *M. enterolobii* had nine *flp* and seven *nlp* orthologous genes to *C. elegans*. All of these gene transcripts were present in *M. enterolobii* and *M. hapla*, except that the *flp-34* gene was absent in *M. enterolobii* (Table S5). However, when we further examined the genes that were involved in neurotransmitter biosynthesis, transport and metabolism, we found that the genes *cha-1*, *ace-3* and *tph-1* that were expressed in *M. hapla* were missing in *M. enterolobii* (Table S6).

#### 2.3.4. RNAi Pathway

The transcripts that were coded for many orthologous proteins to *C. elegans* and participated in siRNA and/or miRNA pathways were also discovered in other nematodes. *M. enterolobii* had similar RNAi machinery to *M. incognita*, *M. hapla* and *G. pallida*. One exception was *mut-7*, which was only found in *M. enterolobii* and *M. hapla*, among all of these nematodes. Compared with *C. elegans*, we failed to find several genes expressed in other nematodes, including *xpo-3*, *smg-5*, *rsd-2* and *sid-1* (Table S7).

#### 2.3.5. Immune Signaling

The immune pathways that were analyzed included TGF-β signaling, ERK MAPK signaling, P39 MAPK signaling and Toll signaling. Using *C. elegans* as the control, we found many orthologous genes from *M. enterolobii*, which were the same as in *M. incognita* (Table S8).

#### 2.3.6. Secretory Proteins

**Figure 5:**
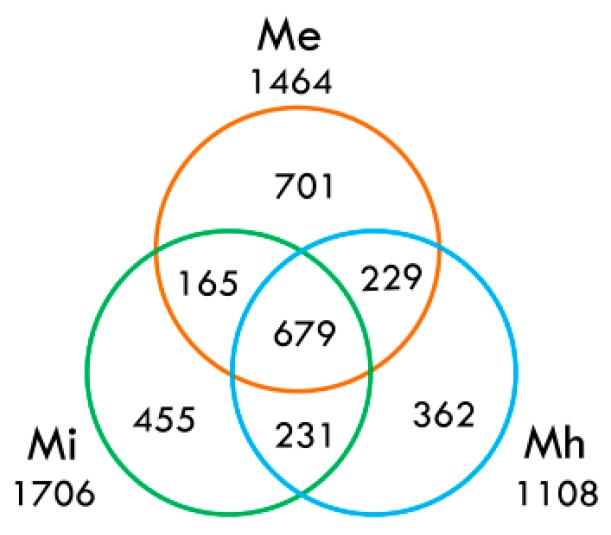
Venn diagram on shared and unique ortholog families of secreted proteins in three *Meloidogyne* species. The numbers in the Venn diagram indicate the ortholog families of secreted proteins. The numbers outside the Venn diagram show the total number of predicted secreted proteins for each *Meloidogyne* species. Me: *M. enterolobii*; Mi: *M. incognita*; Mh: *M. hapla*.

It has been widely accepted that secretions produced by the amphids, hypodermis and esophageal glands of the nematodes involve complicated nematode-plant molecular interactions during the parasitism process. The protein effectors synthesized in esophageal gland cells (dorsal and subventral) and secreted through the stylet into plant cells play essential roles in the parasitism initiation and maintenance especially. From our analysis results, *M. enterolobii* was found to have 1464 putative secretory proteins; *M. incognita* contained as many as 1706; and *M. hapla* only contained 1108 putative secretory proteins. We found 701 predicted secretory proteins that belonged to *M. enterolobii*, 165 belonged to *M. enterolobii* and *M. incognita*; and 229 belonged to *M. enterolobii* and *M. hapla*; of which, 679 were conserved in all three species (Figure 5). Blasting the 1464 putative proteins from *M. enterolobii* showed that 497 of them had orthologous sequences. GO annotations of these proteins from *M. enterolobii* showed that the main functions included cellular parts, i.e., “endoplasmic reticulum-Golgi intermediate compartment”, “vacuole”, “cell periphery”, “extracellular region” and protein folding and proteolysis. In addition to CAZymes, we also identified numerous potential genes that encode secretions; some of which belonged to known parasitic effector homologues that play roles in different key processes, such as: (I) encoding putative chemotaxis participators for host-seeking and location, amphid protein map-1, SXP/RAL-2; (II) encoding plant reprogramming regulators for feeding cells formation and maintenance, expansin (EXP), cellulose-binding proteins (CBP); (III) encoding immunomodulatory components for protecting nematodes against plant defense response, chorismate mutase (CM), fatty acid and retinol binding protein (FAR), venom allergen-like protein (VAP), annexins, etc. Moreover, we also identified some esophageal gland cell secretory proteins that were conserved in plant parasitic nematodes (PPNs), but without functional characterization yet (Table S9).

### 2.4. Expression of the Candidate Genes Suppressed BAX-Induced Cell Death in N. benthamiana

**Figure 6:**
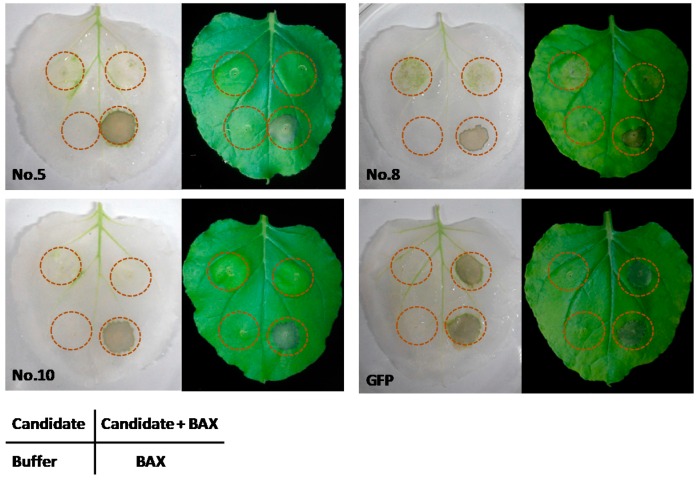
Expression of candidate genes that suppressed BAX-induced programmed cell death (PCD) in *Nicotiana benthamiana*. Four locations of selected leaves (illustrated in the table appended to the figure) were injected with the corresponding solutions. The pictures from the left panel were the leaves that had been boiled in 95% ethanol. No. 5, No. 8 and No. 10 putative proteins suppressed PCD triggered by pro-apoptosis protein BAX, and No. 8 protein also induced necrosis.

Comparing the putative proteins from *M. enterolobii* with the effectors reported, we found 241 transcripts that could be translated into effectors. These putative effectors were involved in cell wall degradation, proteinase, defense or suppression of the host plant immune system, participation in the signaling of the host plants and other similar functions. Of the 241 putative effectors, 75 were biosynthesized from the esophageal gland cells (Table S9). Three candidate secretory proteins were successfully cloned for further analysis. The *Agrobacterium tumefaciens* GV3101 expressing vector pGR107-candidate gene or buffer as a control was injected into the leaves of *N. benthamiana* 24 h before the injection of genes expressing pGR107-BAX. The results showed that the No. 5 and No. 10 proteins could effectively suppress BAX-induced programmed cell death (PCD). Interestingly, the No. 8 protein not only caused necrosis, but also suppressed BAX-induced PCD (Figure 6). The detailed homology information of these three protein sequences is shown in Figure S1.

## 3. Discussion

Our study provides the first transcriptome data of *M. enterolobii*, a pest of economic crop plants. To find out the potential target genes for biological control, we analyzed the transcripts that putatively coded for CAZymes, kinases, neuropeptide genes and secretory proteins and those that were involved in the RNAi pathway and immune signaling, all of which were essential for the successful survival of *M. enterolobii* in its host plants. GO annotation showed that the genes expressed were mainly involved in the structural components and basic metabolic reactions that support their living. The current research studied the nematodes from various stages; therefore, the transcripts that we obtained could provide a generally complete picture of the gene expression pattern of *M. enterolobii*.

We compared the transcripts obtained from *M. enterolobii* with those from five other well-studied nematodes: *C. elegans*, *M. incognita*, *M. hapla*, *G. pallida* and *B. xylophilus*. All of them shared most of their transcriptome information. As expected, *M. enterolobii* had the closest evolutionary relationship with *M. incognita*; next was *M. hapla*; and *M. enterolobii* was evolutionally farthest from *C. elegans*. We analyzed the enriched GO annotated proteins shared by these nematodes. Compared with *M. incognita*, *M. enterolobii* had a similar response to endogenous stimuli, forebrain development and cranial nerve development. The only GO terms shared by *M. hapla* and *M. enterolobii* were enriched in nerve development, B cell activation, brain development, neuron-neuron synaptic transmission, response to cocaine, cation channel activity, response to organic cyclic compounds, learning and responses to ammonium ion. These functions facilitated the survival of these nematodes in reaction to the host plants. Generally, *M. enterolobii* had similar KOs compared with *M. hapla*, although *M. enterolobii* was closest to *M. incognita* evolutionally. However, *M. enterolobii* had its unique expression pattern. For example, the eight unique pathways of *M. enterolobii* (biosynthesis of tetracycline, β-lactam resistance, bisphenol degradation, peptidoglycan biosynthesis, atrazine degradation, steroid degradation, biosynthesis of siderophore group nonribosomal peptides and malaria) may endow *M. incognita* with more competitive ability in host cells. What is worth special mention is malaria (KO_05144), which suggested that *M. enterolobii* may have evolved to have homologous elements involved in the KO_05144 pathway of human malaria parasites, providing clues to reveal the genetic and pathologic mechanism for its strong parasitism ability. Furthermore, *M. incognita* (20,365) had fewer genes expressed overall than did *M. enterolobii* (37,186), but more than *M. hapla* (14,420). This may be due to differences in their living conditions and the evolutionary stress during environmental adaptation.

CAZymes are enzymes that are involved in the synthesis, metabolism and transport of carbohydrates. The first important step for *M. enterolobii* to parasitize a host plant is to destroy the plant cell walls. Glycoside hydrolases are enzymes that can degrade cellulose, which is the main component of plant cell walls. We found 19 putative glycoside hydrolases expressed in *M. enterolobii*, of which 10 putative glycoside hydrolases (GH13, GH18, GH20, GH25, GH30, GH31, GH35, GH37, GH38 and GH84) were expressed in all six nematodes. It should be noted that these genes were expressed in both free-living nematodes (*C. elegans*) and parasitic nematodes, which is possible because GH genes can be acquired via horizontal gene transfer [15].

Protein kinases are another group of influential enzymes that participate in nearly every biochemical reaction. The current research showed that *M. enterolobii* had fewer kinases than *C. elegans* did, especially in the RGC and atypical categories. RGC is an abbreviation for receptor guanylate cyclase, which plays an important role in neuronal signaling systems [16]. The only RGC found in *M. enterolobii* was isotig04408, which was annotated as having multiple functions, such as guanylate cyclase activity, protein tyrosine kinase activity and ATP binding.

Neuropeptides play an essential role in chemoreception in nematodes. To date, 113 genes coding for 250 neuropeptides have been identified in *C. elegans* [17]. Our data showed that *M. enterolobii* and other parasitic root nematodes had many fewer *flp* and *nlp* genes expressed than did *C. elegans*. However, these neuropeptides may still play an important role in their life cycle. For example, the inactivation of *flp-1* can led to many behavioral dysfunctions [18,19].

In the recent ten years, RNAi has been widely employed in functional genomic research on PPNs and has also been perceived as an effective strategy to engineer PPN resistance for host plants. Therefore, identification of the components known to be involved in the RNAi pathway in *M. enterolobii* will be helpful to perform RNAi experiments effectively for this nematode. Different from *C. elegans*, information is limited on the RNAi pathways of these parasitic nematodes. However, we found that most of the genes involved in the RNAi pathway were conserved in these parasitic nematodes, indicating their essential roles in the living cycle of these nematodes. For example, the genes of RNaseIII (e.g., *drsh-1*), RNA helicase (e.g., *drh-3*), export protein (e.g., *xpo-1*) and amplification factors (e.g., *ego-1*) were shown to be expressed in all of the nematodes analyzed.

*M. enterolobii* has a unique immune system in that it avoids other pathogens. For example, *M. enterolobii* can avoid attack from *Pasteuria penetrans*, which is effective for infecting other nematodes [20,21,22]. In the current study, we analyzed the genes that were involved in immune responses of these nematodes by comparison with *C. elegans*, a well-studied nematode model [23,24]. In the TGF-β signaling pathway, *dbl-1* gene was expressed in all six nematodes that were analyzed. The DBL-1 pathway must work with several factors, including the SMA-2/SMA-3/SMA-4 complex and SMA-9 [24]. Any factor missing in the SMA-2/SMA-3/SMA-4 complex will disrupt the downstream cascade [25]. Our data showed that *M. enterolobii*, *M. incognito* and *B. xylophilus* had *sma-2*, *sma-3* and *sma-4* expressed, whereas *M. hapla* and *G. pallida* seemed not to be capable of forming the SMA-2/SMA-3/SMA-4 complex, which might lead to the disruption of this pathway.

Different from other nematodes, *M. enterolobii* can overcome the anti-parasitic genes that are effective in prevention in other nematodes, including the tomato *Mi* resistance gene and pepper *N* resistance gene*.* In addition, *M. enterolobii* could not be infected by *Pasteuria penetrans*, which has potential as a biocontrol agent for *Meloidogyne* species. Therefore, *M. enterolobii* is considered to be a great threat to these crops. Our transcriptome data displayed the unique gene expression pattern of *M. enterolobii* compared with other nematodes. This information may work as a guideline to determine key points (e.g., effectors) for further biological control of these parasites. Despite different lifestyles, the nematodes shared common effectors that enable them to invade root tissues and suppress host immune responses for survival. Esophageal gland cells, including one dorsal gland cell and two subventral gland cells [26], are important organs for the nematodes to synthesize and secrete effectors to interact with the host plants [26,27]. Therefore, identifying the putative proteins at this position has become a widely-used strategy to identify the potential effectors [11,12,13,28,29,30,31,32,33,34,35]. To date, many important effectors secreted from the nematodes have been identified [9,11]. The transcriptomes of many plant parasites have been analyzed using mRNA sequencing, which is an effective technique to discover new effectors [11,12,13]. Jacob et al. used signal peptides and transmembranes as the standard to screen out 156 genes that code for putative effectors [33]. Following these standards, we identified 701 putative secretory proteins that were specifically secreted from esophageal gland cells of *M. enterolobii*. These were many more than those from the other two *Meloidogyne* species, which partly explained why *M. enterolobii* can infect a wide spectrum of host plants.

The pro-apoptotic mouse protein BAX is a death-promoting member of the Bcl-2 family that is involved in programmed cell death (PCD). Because of the common mechanism between the animal and plant cell death program, BAX can also trigger plant PCD (BT-PCD), physiologically resembling that associated with the hypersensitive response [36], so the ability to suppress BT-PCD has proven to be exploited as a valuable initial screening method for pathogen effectors capable of suppressing defense-associated PCD [37,38]. The PPNs’ effectors, which function as immune suppressors, have been postulated to play a decisive role because the successful parasitism was heavily determined by whether or not nematodes and feeding cells keep on surviving without injuries from the plant immune system [39,40,41,42]. In this study, we tested three candidate genes (No. 5, No. 8, No. 10) because they are conserved in *Meloidogyne* genus, and their homologues had been suggested to be exclusively expressed in esophageal gland cells of *M. incognita* by in situ mRNA hybridization [43]. Therefore, it is still logical to deduce that they may be essential genes encoding secretory proteins during the nematode parasitism. In our infiltration assay, No. 5 and No. 10 consistently suppressed BT-PCD, while infiltrating with BAX alone at the same time could initiate a typical BT-PCD reaction (Figure 6), suggesting that No. 5 and No. 10 might be immune modular effectors able to target the common PCD pathways to suppress cell death. Differently, No. 8 protein was able to cause necrosis. This indicated that *M. enterolobii* had multiple strategies to neutralize host immune reactions. To understand their regulation patterns, further research is still needed to confirm the molecular functions of these effectors.

By analyzing the transcriptome data of *M. enterolobii*, we noticed that these parasitic nematodes showed very similar gene expression patterns. Interestingly, these parasitic nematodes missed a variety of genes that were readily expressed in *C. elegans*, which is a free-living nematode. This might be because *C. elegans* was better armed to adapt to the tough free-living environment, whereas the parasitic nematodes were more focused on the defense avoidance of the host plants. It should be noted that the present bioinformation analysis was performed based on *M. enterolobii* expressed sequence tag sequences, which may have led to misidentification because of the potential limitations in transcriptomic coverage and assembly facticity. In the future, the comparative bioinformatics performed on deep/full-length transcriptome or genome sequences, combined with genes’ functional analysis, will facilitate the comprehensive and reliable identification for *M. enterolobii*-specific and *Meloidogyne* conserved genes. Either way, our extensive analyses exposed the specific and conserved genes that are essential to *M. enterolobii* parasitism or survival, and these genes represent the promising targets for novel efficient biocontrol strategies that are friendlier to the environment.

## 4. Materials and Methods

### 4.1. Sample Treatment

*M. enterolobii* stain “MeLD1” was collected from infested tomato roots growing in Ledong, China, whose genetic homogeneity had been identified previously by microscopical observation of perineal patterns [44,45] and by molecular diagnosis using species-specific sequence characterized amplified region (SCAR) markers [5,46]. The genetically homogenous population was multiplied on the susceptible tomato cultivar (*Lycopersicum esculentum* cv. “Baiguo”) in axenic cultures starting from a single-egg mass in a greenhouse of the nematode laboratory in China Agricultural University. Egg masses on root galls were handpicked and hatched over sterile water at 25 °C on improved Baermann pans for 72 h to yield preparasitic second-stage juveniles (pre-J2s). Mixed parasitic stages of *M. enterolobii* were collected from infected tomato roots by root blending and sieving as previous described [47], then the nematodes of the parasitic J2 (para-J2), J3 and adult female stages were separated under microscope based on their morphology differences. All of the collected stages of nematode were frozen (−80 °C) for RNA isolation.

### 4.2. RNA Extraction, cDNA Synthesis and Sequencing

RNA was extracted using TRIzol (Invitrogen, Carlsbad, CA, USA). A Micropoly (A) Purist TM mRNA purification kit (Ambion, Shanghai, China) was used to purify the mRNA under the manufacturer’s instructions. The mRNA from *M. enterolobii* at mixed stages was treated with DNase I at 37 °C for 25 min to remove any residual DNA. The cDNA was then synthesized using SuperScriptTM III Reverse Transcriptase (Invitrogen) at 42 °C for 1 h. The synthesized cDNA was fragmented into 300–800 bp using an ultrasound sonicator (Fisher, Shanghai, China) and purified using Ampure beads (Agencourt, Suzhou, China). The purified cDNA was prepared with a GS DNA Library Preparation kit (Roche Applied Science, Penzberg, Germany), amplified using the GS emPCR kit (Roche Applied Science) and then sequenced in a Roche 454 Genome Sequencer FLX machine.

### 4.3. Assembly and GO Annotation

The reads were assembled using Newbler 2.7 (454 Life sciences, Branford, CT, USA) at the default setting. Genes and associated ORFs were predicted using GetORF from the EMBOSS tool kit and then BLASTed against the Swiss-Prot and TrEMBL databases at an *E* value < 1 × 10^−5^. The functional annotation of all predicted proteins of *M. enterolobii* was performed with Blast2GO [48]. The GO term enrichment analysis was performed using Ontologizer [49] with the term-to-term or parent-child-intersection approach and Bonferroni correction.

### 4.4. Phylogenetic and Comparative Analysis of Orthologous Families

Based on sequence similarity, orthologous families were constructed for predicted proteins of *Meloidogyne enterolobii*, *Meloidogyne* hapla, *Meloidogyne* incognita, *Bursaphelenchus xylophilus*, *Globodera pallida* and *Caenorhabditis elegans* by orthAgogue [50] at the default setting. Protein multiple alignments were carried out using MUSCLE [51] for each of 1022 single-copy ortholog families extracted from the orthAgogue result. The resulting alignments were concatenated into one combined alignment. Poorly-aligned regions were removed by trimAl [52]. The phylogenomic tree was inferred by MEGA6 [53] using the neighbor-joining method with 1000 bootstrap replicates. Using the KEGG Automatic Annotation Server (KAAS) (Available online: http://www.genome.jp/tools/kaas/) with the BBH (bi-directional best hit) method, we mapped the pathways of the three evolutionarily-closest nematodes, i.e., *M. enterolobii*, *M. hapla* and *M. incognita*, and compared the differences in KEGG Ontology (KO) numbers.

### 4.5. Comparative Analysis of the Transcriptome

We compared the transcripts in different nematodes that putatively coded for CAZymes, kinases, neuropeptide genes and secretory proteins and those that were involved in the RNAi pathway and immune signaling. Genes encoding putative carbohydrate-active enzymes were identified using the hmmscan program in the HMMER 3 package [54] to search for the predicted proteomes with the family-specific HMM profiles of CAZymes downloaded from the dbCAN database (Available online: http://csbl.bmb.uga.edu/dbCAN/download/). Kinomes were identified using Kinannote [55]. The neuropeptide-like protein (NLP) and FMRF amide-like peptide (FLP) were identified using BLASTp searches with the known sequences as queries from *Meloidogyne enterolobii*, *Meloidogyne hapla*, *Meloidogyne incognita*, *Bursaphelenchus xylophilus*, *Globodera pallida* and *Caenorhabditis elegans* [56,57,58,59]. RNAi pathway and immune signaling were identified based on the orthAgogue results and the known genes in *C. elegans*. Putative effectors were predicted from the secretory proteins based on two conditions (having signal peptides and not being a membrane protein), as previously reported using SignaIP 4.1 [60] and TMHMM [33].

### 4.6. Functional Verification of Three Putative Effectors

Of the predicted 75 esophageal gland secretory proteins, 3 proteins with signal peptides were cloned. These putative proteins were amplified using the primers listed in Table 1. The PCR product was constructed into the PVX vector pGR107 with a flag-tag fused at the N-terminal and transformed into *A. tumefaciens* GV3101. The verification method was modified from Wang et al. [61]. Briefly, *Agrobacterium* strain GV3101 harboring different constructs was grown in liquid LB medium with suitable antibiotics at 28 °C in a shaking incubator at 200 rpm. After 16 h incubation, the bacterial cells were collected by centrifugation at 5000× *g* for 5 min, washed with 10 mM MgCl_2_ three times and suspended in infiltration medium (10 mM MgCl_2_, 10 mM MES, pH 5.6 and 100 mM acetosyringone) to an OD_600_ of 0.4 and kept at room temperature for 1–3 h prior to infiltration into the four-week-old *N. benthamiana* leaves. The cultured *A. tumefaciens* cells carrying candidate genes and GFP were initially infiltrated into leaves of *N. benthamiana*. After 24 h, the same infiltration site was then challenged with *A. tumefaciens* cells carrying the BAX. The leaves were boiled six days later in 95% ethanol to clear the background for photographing. All experiments were repeated.

## Figures and Tables

**Table 1:** Assembly of the *M. enterolobii* transcriptome.

Assembly Metric	Library	Contigs	Singletons
Total base pairs	165,040,879	–	–
Total reads	408,663	–	–
Reads in assembly	355,760	–	–
Average length (bp)	403	1202	380
EST number	–	8193	31,860
Length range (bp)	–	62–18,104	50–1043
Operon number	–	8143	29,043

EST: Expressed sequence tag.

## Supplementary Materials

Supplementary materials can be found at www.mdpi.com/1422-0067/17/9/1442/s1.
