# Supplementary material for: De Novo Analysis of the Transcriptome of Meloidogyne enterolobii to Uncover Potential Target Genes for Biological Control
Source: Int J Mol Sci. 2016 Sep 1;17(9):1442. doi: 10.3390/ijms17091442 (PMC5037721; doi:10.3390/ijms17091442)
Supplement: Supplementary file 1 [file ijms-17-01442-s001.pdf]

# Supplementary Materials: De Novo Analysis of the Transcriptome of *Meloidogyne enterolobii* to Uncover Potential Target Genes for Biological Control

**Xiangyang Li, Dan Yang, Junhai Niu, Jianlong Zhao and Heng Jian**

**Table S1.** Statistically-significantly enriched GO terms in different categories of orthologous families.

| GO_ID | Name | NSP | *p*-Value | Adj. *p*-Value | Pop. Count | Study Count | Category of Orthologous Families |
|---|---|---|---|---|---|---|---|
| GO:0040028 | regulation of vulval development | B | $5.46 \times 10^{-6}$ | 0.0049 | 38 | 5 | |
| GO:0061062 | regulation of nematode larval development | B | $5.46 \times 10^{-6}$ | 0.0049 | 38 | 5 | |
| GO:0048580 | regulation of post-embryonic development | B | $5.46 \times 10^{-6}$ | 0.0049 | 38 | 5 | |
| GO:0048569 | post-embryonic organ development | B | $1.60 \times 10^{-5}$ | 0.014 | 80 | 6 | |
| GO:2000026 | regulation of multicellular organismal development | B | $3.15 \times 10^{-5}$ | 0.028 | 90 | 6 | Present in all Meloidogyne species |
| GO:0040025 | vulval development | B | $3.47 \times 10^{-5}$ | 0.031 | 55 | 5 | |
| GO:0040027 | negative regulation of vulval development | B | $5.69 \times 10^{-5}$ | 0.051 | 31 | 4 | |
| GO:0061064 | negative regulation of nematode larval development | B | $5.69 \times 10^{-5}$ | 0.051 | 31 | 4 | |
| GO:0048581 | negative regulation of post-embryonic development | B | $5.69 \times 10^{-5}$ | 0.051 | 31 | 4 | |
| GO:0002262 | myeloid cell homeostasis | B | $1.76169 \times 10^{-5}$ | 0.017 | 8 | 3 | |
| GO:0048872 | homeostasis of number of cells | B | $2.62983 \times 10^{-5}$ | 0.026 | 9 | 3 | |
| GO:0009719 | response to endogenous stimulus | B | $2.13189 \times 10^{-5}$ | 0.016 | 99 | 6 | |
| GO:0030900 | forebrain development | B | $5.05432 \times 10^{-7}$ | 0.00063 | 19 | 5 | |
| GO:0021545 | cranial nerve development | B | $6.92617 \times 10^{-7}$ | 0.00087 | 3 | 3 | |
| GO:0021675 | nerve development | B | $2.75299 \times 10^{-6}$ | 0.0034 | 4 | 3 | |
| GO:0042113 | B cell activation | B | $6.83907 \times 10^{-6}$ | 0.0086 | 5 | 3 | |
| GO:0007420 | brain development | B | $1.09443 \times 10^{-5}$ | 0.014 | 34 | 5 | |
| GO:0007270 | neuron-neuron synaptic transmission | B | $1.35919 \times 10^{-5}$ | 0.017 | 6 | 3 | |
| GO:0042220 | response to cocaine | B | $3.75791 \times 10^{-5}$ | 0.047 | 8 | 3 | |
| GO:0005261 | cation channel activity | M | $4.37561 \times 10^{-5}$ | 0.055 | 149 | 8 | |
| GO:0014070 | response to organic cyclic compound | B | $5.21082 \times 10^{-5}$ | 0.065 | 76 | 6 | |
| GO:0007612 | learning | B | $5.60136 \times 10^{-5}$ | 0.070 | 9 | 3 | |
| GO:0060359 | response to ammonium ion | B | $7.95154 \times 10^{-5}$ | 0.099 | 10 | 3 | |

GO ID: the accession number of the GO term; Name: the name of the GO term.; NSP: the sub-ontology: biological process (B), cellular component (C) or molecular function (M); *p*-value: the nominal (uncorrected) *p*-value resulting from the observed overrepresentation of the GO term Adj. *p*-Value: the adjusted *p*-Value (adjusted by the MTC procedure chosen by the user); Pop. Count: the number of genes in the population set that are annotated to the GO term in question; Study Count: The number of genes in the study set that are annotated to the GO term in question.

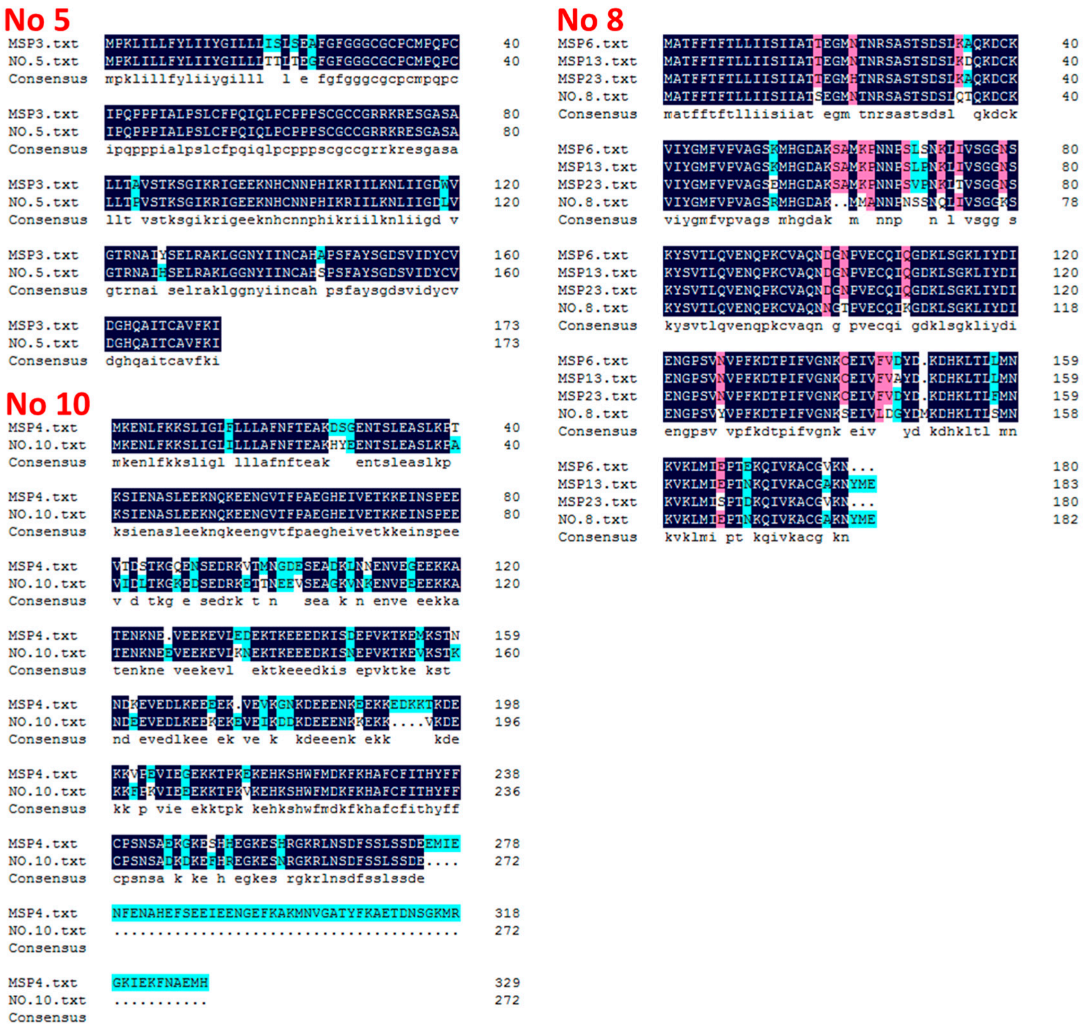


**Figure S1.** Predicted protein sequence and homology of the candidate effectors. By the comparison using NCBI, No. 5 protein had high homology with the putative esophageal gland cell secretory protein 3 (Meloidogyne incognita); No. 8 protein had high homology with the putative esophageal gland cell secretory protein 13, putative esophageal gland cell secretory protein 6 and putative esophageal gland cell secretory protein 23 (*Meloidogyne incognita*); while No. 10 had high homology with the putative esophageal gland cell secretory protein 4 (*Meloidogyne incognita*).

**Table S2.** Different numbers of the KEGG Ontology (KO) predicted for *M. enterolobii*, *M. hapla* and *M. incognita*.

| KO_ID | Pathway | Mi_No. | Mh_No. | Me_No. |
|---|---|---|---|---|
| 00760 | Nicotinate and nicotinamide metabolism | 8 | 8 | 12 |
| 00720 | Carbon fixation pathways in prokaryotes | 8 | 8 | 10 |
| 00561 | Glycerolipid metabolism | 12 | 14 | 17 |
| 00253 | Tetracycline biosynthesis | 0 | 0 | 1 |
| 00312 | beta-Lactam resistance | 0 | 0 | 1 |
| 00363 | Bisphenol degradation | 0 | 0 | 1 |
| 00550 | Peptidoglycan biosynthesis | 0 | 0 | 1 |
| 00791 | Atrazine degradation | 0 | 0 | 1 |
| 00984 | Steroid degradation | 0 | 0 | 1 |
| 01053 | Biosynthesis of siderophore group nonribosomal peptides | 0 | 0 | 1 |
| 05144 | Malaria | 0 | 0 | 1 |
| 00332 | Carbapenem biosynthesis | 2 | 0 | 0 |
| 00906 | Carotenoid biosynthesis | 1 | 0 | 0 |
| 00941 | Flavonoid biosynthesis | 1 | 0 | 0 |
| 00942 | Anthocyanin biosynthesis | 1 | 0 | 0 |
| 00945 | Stilbenoid, diarylheptanoid and gingerol biosynthesis | 1 | 0 | 0 |
| 05321 | Inflammatory bowel disease (IBD) | 0 | 1 | 0 |
| 05016 | Huntington's disease | 0 | 86 | 83 |
| 05169 | Epstein-Barr virus infection | 0 | 65 | 63 |
| 04932 | Non-alcoholic fatty liver disease (NAFLD) | 0 | 58 | 61 |
| 05166 | Human T-Cell lymphotropic viruse I (HTLV-I) infection | 0 | 64 | 60 |
| 05168 | Herpes simplex infection | 0 | 35 | 38 |
| 05152 | Tuberculosis | 0 | 31 | 31 |
| 05034 | Alcoholism | 0 | 31 | 29 |
| 05100 | Bacterial invasion of epithelial cells | 0 | 32 | 29 |
| 05110 | Vibrio cholerae infection | 0 | 29 | 28 |
| 05120 | Epithelial cell signaling in *Helicobacter pylori* infection | 0 | 24 | 28 |
| 05161 | Hepatitis B | 0 | 27 | 26 |
| 05164 | Influenza A | 0 | 25 | 25 |
| 05132 | Salmonella infection | 0 | 23 | 23 |
| 05131 | Shigellosis | 0 | 25 | 21 |

**Table S2.** *Cont.*

| KO_ID | Pathway | Mi_No. | Mh_No. | Me_No. |
|---|---|---|---|---|
| 05160 | Hepatitis C | 0 | 18 | 19 |
| 05032 | Morphine addiction | 0 | 21 | 18 |
| 05162 | Measles | 0 | 15 | 18 |
| 05142 | Chagas disease (American trypanosomiasis) | 0 | 16 | 17 |
| 05145 | Toxoplasmosis | 0 | 17 | 17 |
| 05130 | Pathogenic Escherichia coli infection | 0 | 17 | 16 |
| 05146 | Amoebiasis | 0 | 16 | 16 |
| 05031 | Amphetamine addiction | 0 | 15 | 15 |
| 05134 | Legionellosis | 0 | 12 | 13 |
| 05030 | Cocaine addiction | 0 | 12 | 12 |
| 04930 | Type II diabetes mellitus | 0 | 9 | 11 |
| 05410 | Hypertrophic cardiomyopathy (HCM) | 0 | 10 | 11 |
| 05414 | Dilated cardiomyopathy (DCM) | 0 | 10 | 11 |
| 05412 | Arrhythmogenic right ventricular cardiomyopathy | 0 | 8 | 9 |
| 05020 | Prion diseases | 0 | 9 | 8 |
| 05133 | Pertussis | 0 | 8 | 8 |
| 05416 | Viral myocarditis | 0 | 7 | 7 |
| 04950 | Maturity onset diabetes of the young | 0 | 6 | 6 |
| 05033 | Nicotine addiction | 0 | 4 | 4 |
| 05140 | Leishmaniasis | 0 | 5 | 4 |
| 00290 | Valine, leucine and isoleucine biosynthesis | 0 | 2 | 3 |
| 04940 | Type I diabetes mellitus | 0 | 4 | 3 |
| 00300 | Lysine biosynthesis | 0 | 1 | 2 |
| 05143 | African trypanosomiasis | 0 | 3 | 2 |
| 00254 | Aflatoxin biosynthesis | 0 | 1 | 1 |
| 00626 | Naphthalene degradation | 0 | 1 | 1 |
| 01220 | Degradation of aromatic compounds | 0 | 1 | 1 |

Me: M. enterolobii; Mi: M. incognito; Mh: M. hapla.

**Table S3.** The numbers of CAZyme modules or domains in different nematodes.

| CAZy Family | Bx | Ce | Gp | Me | Mh | Mi | CAZy Family | Bx | Ce | Gp | Me | Mh | Mi | CAZy Family | Bx | Ce | Gp | Me | Mh | Mi |
|---|---|---|---|---|---|---|---|---|---|---|---|---|---|---|---|---|---|---|---|---|
| AA3 | 1 | 1 | 1 | 1 | 1 | 2 | GH20 | 5 | 5 | 2 | 2 | 3 | 4 | GT24 | 2 | 2 | 0 | 1 | 1 | 0 |
| AA4 | 2 | 3 | 0 | 1 | 1 | 2 | GH22 | 0 | 0 | 0 | 0 | 0 | 1 | GT25 | 1 | 1 | 1 | 1 | 1 | 1 |
| AA7 | 1 | 0 | 8 | 0 | 0 | 1 | GH25 | 3 | 10 | 1 | 10 | 5 | 5 | GT26 | 2 | 0 | 0 | 0 | 0 | 0 |
| CBM13 | 9 | 8 | 5 | 7 | 7 | 7 | GH27 | 3 | 1 | 0 | 1 | 1 | 1 | GT27 | 11 | 9 | 10 | 9 | 8 | 9 |
| CBM14 | 19 | 46 | 20 | 27 | 22 | 8 | GH28 | 0 | 0 | 0 | 0 | 4 | 4 | GT28 | 1 | 1 | 1 | 0 | 1 | 4 |
| CBM18 | 0 | 7 | 0 | 0 | 0 | 0 | GH29 | 3 | 1 | 0 | 1 | 1 | 2 | GT3 | 2 | 1 | 1 | 1 | 1 | 0 |
| CBM2 | 0 | 0 | 12 | 4 | 4 | 13 | GH3 | 0 | 0 | 1 | 0 | 0 | 0 | GT31 | 35 | 31 | 33 | 10 | 17 | 22 |
| CBM20 | 2 | 3 | 1 | 2 | 2 | 1 | GH30 | 9 | 4 | 14 | 5 | 4 | 8 | GT33 | 1 | 1 | 1 | 1 | 1 | 2 |
| CBM21 | 1 | 2 | 5 | 1 | 1 | 2 | GH31 | 4 | 4 | 5 | 3 | 3 | 1 | GT34 | 1 | 2 | 2 | 3 | 1 | 4 |
| CBM29 | 0 | 0 | 0 | 1 | 0 | 1 | GH32 | 0 | 0 | 11 | 2 | 2 | 2 | GT35 | 2 | 1 | 1 | 2 | 1 | 3 |
| CBM32 | 7 | 4 | 5 | 5 | 4 | 3 | GH35 | 1 | 2 | 2 | 2 | 1 | 4 | GT39 | 2 | 0 | 0 | 0 | 0 | 0 |
| CBM37 | 1 | 0 | 0 | 0 | 0 | 0 | GH37 | 6 | 5 | 15 | 4 | 5 | 5 | GT4 | 5 | 4 | 9 | 4 | 6 | 9 |
| CBM40 | 0 | 0 | 0 | 0 | 0 | 1 | GH38 | 5 | 3 | 3 | 3 | 3 | 1 | GT41 | 2 | 3 | 1 | 1 | 3 | 1 |
| CBM48 | 1 | 2 | 0 | 1 | 2 | 3 | GH43 | 0 | 0 | 1 | 0 | 2 | 2 | GT43 | 12 | 7 | 5 | 2 | 1 | 1 |
| CBM49 | 1 | 0 | 0 | 0 | 0 | 0 | GH45 | 11 | 0 | 0 | 0 | 0 | 0 | GT44 | 0 | 0 | 1 | 1 | 0 | 0 |
| CBM50 | 0 | 11 | 0 | 2 | 4 | 3 | GH47 | 7 | 7 | 8 | 6 | 6 | 8 | GT47 | 2 | 1 | 6 | 1 | 2 | 1 |
| CBM52 | 0 | 0 | 0 | 0 | 1 | 0 | GH5 | 0 | 0 | 15 | 8 | 10 | 31 | GT49 | 11 | 10 | 0 | 0 | 0 | 0 |
| CBM56 | 0 | 0 | 1 | 0 | 0 | 0 | GH53 | 0 | 0 | 1 | 0 | 0 | 0 | GT50 | 1 | 1 | 2 | 0 | 0 | 0 |
| CBM57 | 0 | 1 | 0 | 0 | 0 | 0 | GH56 | 3 | 1 | 0 | 0 | 1 | 2 | GT54 | 1 | 0 | 1 | 0 | 1 | 0 |
| CBM62 | 0 | 1 | 0 | 0 | 0 | 0 | GH59 | 0 | 1 | 0 | 0 | 0 | 0 | GT57 | 2 | 2 | 1 | 2 | 2 | 1 |
| CBM63 | 0 | 1 | 1 | 0 | 0 | 0 | GH63 | 1 | 1 | 0 | 2 | 1 | 3 | GT58 | 1 | 1 | 1 | 0 | 2 | 0 |
| CBM66 | 2 | 3 | 1 | 0 | 0 | 0 | GH75 | 0 | 1 | 0 | 2 | 1 | 1 | GT59 | 2 | 2 | 2 | 0 | 1 | 2 |
| CE1 | 17 | 24 | 8 | 11 | 7 | 16 | GH76 | 0 | 1 | 0 | 0 | 1 | 0 | GT61 | 0 | 1 | 0 | 0 | 0 | 0 |
| CE10 | 50 | 56 | 20 | 10 | 17 | 17 | GH82 | 0 | 0 | 0 | 0 | 1 | 0 | GT64 | 6 | 1 | 4 | 1 | 1 | 0 |
| CE14 | 1 | 1 | 0 | 0 | 1 | 1 | GH84 | 1 | 1 | 1 | 1 | 1 | 1 | GT65 | 1 | 1 | 2 | 0 | 0 | 0 |
| CE4 | 1 | 2 | 1 | 0 | 0 | 0 | GH85 | 1 | 1 | 0 | 1 | 1 | 1 | GT66 | 2 | 1 | 2 | 2 | 2 | 4 |
| CE5 | 4 | 3 | 0 | 0 | 0 | 0 | GH89 | 0 | 1 | 0 | 0 | 0 | 0 | GT68 | 2 | 1 | 1 | 1 | 1 | 1 |
| CE7 | 4 | 4 | 0 | 0 | 0 | 0 | GH9 | 1 | 0 | 0 | 0 | 0 | 0 | GT7 | 4 | 4 | 7 | 6 | 8 | 12 |
| CE9 | 0 | 1 | 0 | 1 | 1 | 0 | GH99 | 2 | 0 | 1 | 0 | 0 | 0 | GT75 | 0 | 10 | 0 | 0 | 0 | 0 |
| GH1 | 1 | 2 | 0 | 0 | 0 | 0 | GT1 | 43 | 69 | 19 | 10 | 15 | 30 | GT76 | 1 | 1 | 1 | 0 | 1 | 1 |
| GH104 | 0 | 0 | 2 | 0 | 0 | 0 | GT10 | 16 | 5 | 16 | 5 | 13 | 26 | GT77 | 3 | 6 | 2 | 1 | 3 | 3 |

**Table S3.** *Cont.*

| CAZy Family | Bx | Ce | Gp | Me | Mh | Mi | CAZy Family | Bx | Ce | Gp | Me | Mh | Mi | CAZy Family | Bx | Ce | Gp | Me | Mh | Mi |
|---|---|---|---|---|---|---|---|---|---|---|---|---|---|---|---|---|---|---|---|---|
| GH109 | 7 | 0 | 2 | 3 | 1 | 1 | GT11 | 2 | 25 | 6 | 5 | 11 | 16 | GT8 | 3 | 6 | 4 | 4 | 1 | 2 |
| GH116 | 1 | 2 | 0 | 0 | 0 | 0 | GT13 | 2 | 4 | 2 | 0 | 2 | 5 | GT83 | 0 | 0 | 2 | 0 | 0 | 0 |
| GH120 | 0 | 0 | 0 | 0 | 1 | 1 | GT14 | 15 | 20 | 16 | 12 | 10 | 7 | GT84 | 1 | 0 | 0 | 0 | 0 | 0 |
| GH128 | 0 | 1 | 0 | 0 | 0 | 0 | GT16 | 2 | 1 | 2 | 2 | 1 | 0 | GT87 | 0 | 1 | 0 | 0 | 0 | 0 |
| GH13 | 5 | 5 | 2 | 2 | 4 | 3 | GT18 | 1 | 1 | 0 | 0 | 0 | 0 | GT92 | 14 | 61 | 18 | 1 | 2 | 1 |
| GH15 | 2 | 2 | 0 | 0 | 0 | 0 | GT2 | 4 | 5 | 4 | 3 | 4 | 3 | PL14 | 0 | | 1 | 0 | 1 | 0 |
| GH16 | 8 | 0 | 0 | 0 | 0 | 0 | GT20 | 1 | 2 | 3 | 1 | 1 | 8 | PL22 | 1 | 1 | 0 | 0 | 0 | 0 |
| GH18 | 12 | 39 | 11 | 6 | 9 | 3 | GT21 | 1 | 3 | 1 | 1 | 1 | 0 | PL3 | 15 | 0 | 8 | 11 | 20 | 33 |
| GH19 | 2 | 4 | 2 | 0 | 1 | 4 | GT22 | 3 | 3 | 3 | 2 | 3 | 2 | | | | | | | |
| GH2 | 4 | 2 | 1 | 0 | 2 | 5 | GT23 | 1 | 1 | 1 | 4 | 9 | 19 | Total | 475 | 606 | 393 | 249 | 310 | 418 |

Ce: *C. elegans*; Me: *M. enterolobii*; Mi: *M. incognito*; Mh: *M. hapla*; Gp: *G. pallida*; Bx: *B. xylophilus*. AA: auxiliary activities; CBM: carbohydrate binding module; CE: carbohydrate esterase; GH: glycoside hydrolases; GT: glycosyltransferase; PL: polysaccharide lyase.

**Table S4.** Comparison of the kinomes in selected nematode genomes.

| Kinase-Group | Bx | Ce | Gp | Me | Mh | Mi |
|---|---|---|---|---|---|---|
| **Total** | **376** | **456** | **229** | **169** | **238** | **374** |
| AGC | 28 | 30 | 19 | 17 | 20 | 26 |
| CAMK | 34 | 39 | 34 | 18 | 24 | 39 |
| CK1 | 49 | 69 | 10 | 14 | 18 | 45 |
| CMGC | 40 | 49 | 34 | 23 | 30 | 40 |
| Other | 43 | 46 | 32 | 19 | 22 | 29 |
| RGC | 8 | 27 | 8 | 1 | 13 | 14 |
| STE | 25 | 24 | 16 | 11 | 14 | 22 |
| TK | 65 | 79 | 20 | 14 | 22 | 26 |
| TKL | 15 | 14 | 11 | 6 | 7 | 12 |
| twilight | 46 | 47 | 15 | 32 | 44 | 59 |
| unclassified | 20 | 26 | 26 | 12 | 20 | 52 |
| Atypical | 3 | 6 | 4 | 2 | 4 | 10 |

Ce: *C. elegans*; Me: *M. enterolobii*; Mi: *M. incognito*; Mh: *M. hapla*; Gp: *G. pallida*; Bx: *B. xylophilus*.

**Table S5.** Presence of *flp* and *nlp* neuropeptide-encoding genes in *M. enterolobii* and other nematodes.

| Ce | Mi | Mh | Me | Gp | Bx |
|---|---|---|---|---|---|
| *flp*-1 | + | + | + | | + |
| *flp*-6 | | + | + | + | + |
| *flp*-11 | | + | + | | + |
| *flp*-12 | + | + | + | + | + |
| *flp*-13 | | | | | + |
| *flp*-14 | + | | + | | + |
| *flp*-16 | + | + | + | + | |
| *flp*-17 | | + | + | + | + |
| *flp*-18 | | + | + | | + |
| *flp*-22 | + | + | + | | + |
| *flp*-34 | + | + | | + | + |
| *nlp*-1 | + | + | + | | |
| *nlp*-2 | + | + | + | | + |
| *nlp*-3 | | | | | + |
| *nlp*-10 | + | + | + | + | + |
| *nlp*-12 | + | + | + | + | |
| *nlp*-14 | | | | | + |
| *nlp*-15 | + | + | + | + | |
| *nlp*-21 | | + | + | + | + |
| *nlp*-37 | | + | + | | + |
| *nlp*-40 | | | | | + |
| *nlp*-42 | | | | + | + |

Ce: *C. elegans*; Me: *M. enterolobii*; Mi: *M. incognito*; Mh: *M. hapla*; Gp: *G. pallida*; Bx: *B. xylophilus*.

**Table S6.** Presence of neurotransmitter biosynthesis, transport and metabolism genes in *M. enterolobii* and other nematodes.

| | **Gene Function** | **Ce** | **Mi** | **Mh** | **Me** | **Gp** | **Bx** |
|---|---|---|---|---|---|---|---|
| Acetylcholine | choline acetyltransferase | *cha*-1 | + | + | | + | + |
| | synaptic acetylcholine transporter | *unc*-17 | | + | + | + | + |
| | choline transporter | *cho*-1 | | + | + | + | + |
| | post-synaptic transporter | *snf*-6 | + | + | + | + | + |
| | acetylcholinesterase | *ace*-1 | | + | + | + | + |
| | acetylcholinesterase | *ace*-2 | + | + | + | + | + |
| | acetylcholinesterase | *ace*-3 | + | + | | + | + |
| | acetylcholinesterase | *ace*-4 | | | | | |
| Serotonin | tryptophan hydroxylase | *tph*-1 | | + | | + | + |
| | GTP-cyclohydrolase I | *cat*-4 | | + | + | + | + |
| | aromatic AA decarboxylase | *bas*-1 | | + | + | + | + |
| | vesicular monoamine transporter | *cat*-1 | | + | + | + | + |
| | serotonin reuptake transporter | *mod*-5 | + | + | + | + | + |
| | monoamine oxidase | *amx*-1 | | | | | + |
| | monoamine oxidase | *amx*-2 | | | | | |
| | monoamine oxidase | *amx*-3 | | | | | |
| Dopamine | tyrosine hydroxylase | *cat*-2 | | | | | + |
| | dopamine reuptake transporter | *dat*-1 | | + | | + | + |
| Tyramine | tyrosine decarboxylase | *tdc*-1 | + | + | + | + | + |
| Octopamine | tyramine β-hydroxylase | *tbh*-1 | + | + | + | | + |
| Glutamate | vesicular glutamate transporter | *eat*-4 | + | + | + | + | + |
| | plasma membrane glutamate transporter | *glt*-1 | + | + | + | + | + |
| GABA | glutamate decarboxylase | *unc*-25 | + | + | + | + | + |
| | vesicular GABA transporter | *unc*-47 | + | + | + | + | + |
| | GABA transporter | *snf*-11 | + | + | + | + | + |
| | GABA transaminase | *gta*-1 | + | + | + | + | + |

Ce: *C. elegans*; Me: *M. enterolobii*; Mi: *M. incognito*; Mh: *M. hapla*; Gp: *G. pallida*; Bx: *B. xylophilus*.

**Table S7.** Comparison of genes involved in the RNAi pathway in *M. enterolobii* and other nematodes.

| RNAi Pathway | Ce | Mi | Mh | Me | Gp | Bx |
|---|---|---|---|---|---|---|
| **Small RNA biosynthetic proteins** | *drh*-3 | + | + | + | + | + |
| | *drsh*-1 | + | + | + | + | + |
| | *xpo*-1 | + | + | + | + | + |
| | *xpo*-2 | + | + | + | + | + |
| | *dcr*-1 | + | + | + | + | + |
| | *drh*-1 | + | + | + | | + |
| | *pash*-1 | + | + | + | + | + |
| | *rde*-4 | | | | | + |
| | *xpo*-3 | | | | | |
| **Amplification** | *smg*-2 | + | + | + | + | + |
| | *smg*-6 | + | + | + | + | + |
| | *ego*-1 | + | + | + | + | + |
| | *rrf*-3 | | | | | + |
| | *rrf*-1 | + | + | + | + | + |
| | *smg*-5 | | | | | |
| | *rsd*-2 | | | | | |
| **Spreading** | *rsd*-3 | + | + | + | + | + |
| | *sid*-1 | | | | | |
| | *rsd*-6 | | | | | |
| | *sid*-2 | | | | | |

**Table S7.** *Cont.*

| RNAi Pathway | Ce | Mi | Mh | Me | Gp | Bx |
|---|---|---|---|---|---|---|
| **Argonautes** | *alg*-1 | + | + | + | + | + |
| | R06C7.1 | + | + | + | + | + |
| | C04F12.1 | | | | | |
| | F58G1.1 | + | + | + | + | + |
| | *alg*-4 | | | | | + |
| | *rde*-1 | | | | | |
| | C16C10.3 | + | + | + | + | + |
| | *ppw*-1 | | | | | |
| | *csr*-1 | + | + | + | + | + |
| | *ppw*-2 | + | + | + | + | + |
| | *sago*-1 | | | | | |
| | T22B3.2 | | | | | + |
| | T22H9.3 | + | + | + | + | + |
| | alg-2 | + | + | + | + | + |
| | *ergo*-1 | | | | | |
| | *prg*-1 | | | | | |
| | F55A12.1 | | | | | |
| | T23D8.7 | | | | | |
| | *nrde*-3 | + | + | + | + | + |
| | *sago*-2 | | | | | |
| | T23B3.2 | | | | | |
| | Y49F6A.1 | + | + | + | + | + |
| | ZK1248.7 | + | + | + | + | + |
| | *prg*-2 | | | | | |
| **Other RISC components** | *tsn*-1 | + | + | + | + | + |
| | *ain*-1 | | | | | |
| | *vig*-1 | | | | | + |
| | *ain*-2 | | | | | |

**Table S7.** *Cont.*

| RNAi Pathway | Ce | Mi | Mh | Me | Gp | Bx |
|---|---|---|---|---|---|---|
| **RNAi inhibitors** | *eri*-1 | + | + | + | + | |
| | *xrn*-2 | + | + | + | + | + |
| | *adr*-2 | | | | | |
| | *xrn*-1 | | | | | |
| | *adr*-1 | + | | | | + |
| | *lin*-15b | | | | | |
| | *eri*-5 | + | + | + | + | + |
| | *eri*-6 | | | | | |
| | *eri*-7 | | | | | + |
| | *eri*-3 | | | | | |
| **Nuclear RNAi effectors** | *mut*-7 | | + | + | | + |
| | *cid*-1 | + | + | + | + | + |
| | *ekl*-1 | + | + | + | + | + |
| | *gfl*-1 | + | | + | + | + |
| | *mes*-2 | + | + | + | + | + |
| | *ekl*-4 | + | + | + | | + |
| | *mes*-6 | | + | + | + | + |
| | *rha*-1 | + | + | + | + | + |
| | *ekl*-6 | | | | | + |
| | *zfp*-1 | | | | | |
| | *mut*-2 | | | | | |
| | *ekl*-5 | | | | | |
| | *mes*-3 | | | | | |
| | *mut*-16 | | | | | |
| | *rde*-2 | | | | | |

Ce: *C. elegans*; Me: *M. enterolobii*; Mi: *M. incognito*; Mh: *M. hapla*; Gp: *G. pallida*; Bx: *B. xylophilus*.

**Table S8.** Presence of genes involved in immune signaling in *M. enterolobii* and other nematodes.

| Immune signaling | Ce | Mi | Mh | Me | Gp | Bx |
|---|---|---|---|---|---|---|
| TGF-β signaling pathway | *dbl*-1 | + | + | + | + | + |
| | *sma*-2 | + | | + | + | + |
| | *sma*-3 | + | + | + | | + |
| | *sma*-4 | + | | + | | + |
| ERK MAPK signaling pathway | *lin*-45 | + | + | + | + | + |
| | *mak*-2 | + | + | + | + | + |
| | *mpk*-1 | + | + | + | + | + |
| P39 MAPK signaling pathway | *nsy*-1 | + | + | + | + | + |
| | *pmk*-1 | | + | | + | + |
| | *sek*-1 | + | + | + | + | + |
| | *tir*-1 | + | + | + | + | + |
| Toll signaling pathway | *tol*-1 | + | + | + | + | + |
| | *trf*-1 | | | | | + |
| | *ikb*-1 | | | | | |
| | *plk*-1 | + | + | + | + | + |

Ce: *C. elegans*; Me: *M. enterolobii*; Mi: *M. incognito*; Mh: *M. hapla*; Gp: *G. pallida*; Bx: *B. xylophilus*.

**Table S9.** Putative secretory proteins of *M. enterolobii*.

| Gene Name | *E* Value | ID | Function | Species |
|---|---|---|---|---|
| *IK2WLD401ASNAP_5* | $3 \times 10^{-33}$ | gb\|AAQ10016.1 | putative esophageal gland cell secretory protein 2 | *Meloidogyne incognita* |
| *IK2WLD401BNOW1_7* | $4 \times 10^{-24}$ | gb\|AAN08591.1 | putative esophageal gland cell secretory protein 25 | *Meloidogyne incognita* |
| *IK2WLD401CC5IY_3* | $5 \times 10^{-45}$ | gb\|AAF76925.1 | AF159590_1 hypothetical esophageal gland cell secretory protein 11 | *Heterodera glycines* |
| *IK2WLD401CM1KN_10* | $1 \times 10^{-16}$ | gb\|AAN08583.1 | putative esophageal gland cell secretory protein 17 | *Meloidogyne incognita* |
| *IK2WLD401D16V8_8* | $1 \times 10^{-33}$ | gb\|AAN08583.1 | putative esophageal gland cell secretory protein 17 | *Meloidogyne incognita* |
| *IK2WLD401DDL8G_8* | $7 \times 10^{-53}$ | gb\|AAF76926.1 | AF159591_1 hypothetical esophageal gland cell secretory protein 12 | *Heterodera glycines* |
| *IK2WLD401DNW43_3* | $4 \times 10^{-36}$ | gb\|AAF76925.1 | AF159590_1 hypothetical esophageal gland cell secretory protein 11 | *Heterodera glycines* |
| *IK2WLD401DXTOX_7* | $1 \times 10^{-53}$ | gb\|AAN08585.1 | putative esophageal gland cell secretory protein 19 | *Meloidogyne incognita* |
| *IK2WLD401EBWBC_5* | $3 \times 10^{-23}$ | gb\|AAQ10017.1 | putative esophageal gland cell secretory protein 3 | *Meloidogyne incognita* |
| *IK2WLD402FVOYL_5* | $1 \times 10^{-13}$ | gb\|AAQ10017.1 | putative esophageal gland cell secretory protein 3 | *Meloidogyne incognita* |
| *IK2WLD402FXA8R_5* | $6 \times 10^{-21}$ | gb\|AAN15808.1 | putative esophageal gland cell secretory protein 28 | *Meloidogyne incognita* |

**Table S9.** *Cont.*

| Gene Name | *E* Value | ID | Function | Species |
|---|---|---|---|---|
| *IK2WLD402HJ8JG_9* | $2 \times 10^{-7}$ | gb\|AAK94492.1 | hypothetical esophageal gland protein scn1144 | *Heterodera glycines* |
| *IK2WLD402HQP79_5* | $5 \times 10^{-63}$ | gb\|AAN52090.1 | putative esophageal gland cell secretory protein 32 | *Meloidogyne incognita* |
| *IK2WLD402IGCSD_7* | $4 \times 10^{-27}$ | gb\|AAN08591.1 | putative esophageal gland cell secretory protein 25 | *Meloidogyne incognita* |
| *IKGN5RE01A3VIH_2* | $5 \times 10^{-11}$ | gb\|AAN15809.1 | putative esophageal gland cell secretory protein 29 | *Meloidogyne incognita* |
| *IKGN5RE01A74GJ_2* | $2 \times 10^{-52}$ | gb\|AAF76926.1 | AF159591_1 hypothetical esophageal gland cell secretory protein 12 | *Heterodera glycines* |
| *IKGN5RE01A81OV_8* | $4 \times 10^{-47}$ | gb\|AAF76926.1 | AF159591_1 hypothetical esophageal gland cell secretory protein 12 | *Heterodera glycines* |
| *IKGN5RE01A8FU5_7* | $2 \times 10^{-10}$ | gb\|AAF76926.1 | AF159591_1 hypothetical esophageal gland cell secretory protein 12 | *Heterodera glycines* |
| *IKGN5RE01AJLMG_1* | $2 \times 10^{-7}$ | gb\|AAN15808.1 | putative esophageal gland cell secretory protein 28 | *Meloidogyne incognita* |
| *IKGN5RE01AO2FR_2* | $2 \times 10^{-52}$ | gb\|AAF76926.1 | AF159591_1 hypothetical esophageal gland cell secretory protein 12 | *Heterodera glycines* |
| *IKGN5RE01AQNIQ_2* | $7 \times 10^{-28}$ | gb\|AAN08585.1 | putative esophageal gland cell secretory protein 19 | *Meloidogyne incognita* |
| *IKGN5RE01AXQ9P_9* | $4 \times 10^{-30}$ | gb\|AAQ10020.1 | putative esophageal gland cell secretory protein 6 | *Meloidogyne incognita* |
| *IKGN5RE01B2CCZ_7* | $3 \times 10^{-38}$ | gb\|AAN08591.1 | putative esophageal gland cell secretory protein 25 | *Meloidogyne incognita* |
| *IKGN5RE01BFIYU_5* | $4 \times 10^{-39}$ | gb\|AAF76926.1 | AF159591_1 hypothetical esophageal gland cell secretory protein 12 | *Heterodera glycines* |
| *IKGN5RE01BGZCY_2* | $6 \times 10^{-25}$ | gb\|AAQ10020.1 | putative esophageal gland cell secretory protein 6 | *Meloidogyne incognita* |
| *IKGN5RE01BLHBU_8* | $4 \times 10^{-25}$ | gb\|AAN52095.1 | putative esophageal gland cell secretory protein 31 | *Meloidogyne incognita* |
| *IKGN5RE01BSRIR_7* | $5 \times 10^{-8}$ | gb\|AAF76926.1 | AF159591_1 hypothetical esophageal gland cell secretory protein 12 | *Heterodera glycines* |
| *IKGN5RE01BV957_3* | $2 \times 10^{-6}$ | gb\|AAN08587.1 | putative esophageal gland cell secretory protein 21 | *Meloidogyne incognita* |
| *IKGN5RE01BV9AQ_6* | $2 \times 10^{-40}$ | gb\|AAF76926.1 | AF159591_1 hypothetical esophageal gland cell secretory protein 12 | *Heterodera glycines* |
| *IKGN5RE01C1JO4_2* | $5 \times 10^{-29}$ | gb\|AAN08591.1 | putative esophageal gland cell secretory protein 25 | *Meloidogyne incognita* |
| *IKGN5RE01C6CPD_6* | $1 \times 10^{-52}$ | gb\|AAQ10020.1 | putative esophageal gland cell secretory | *Meloidogyne incognita* |
| *IKGN5RE01CANMP_10* | $6 \times 10^{-63}$ | gb\|AAN08586.1 | putative esophageal gland cell secretory protein 20 | *Meloidogyne incognita* |
| *IKGN5RE01CMMZB_1* | $9 \times 10^{-24}$ | gb\|AAN15807.1 | putative esophageal gland cell secretory protein 27 | *Meloidogyne incognita* |
| *IKGN5RE01CQVXJ_3* | $3 \times 10^{-8}$ | gb\|AAF76926.1 | AF159591_1 hypothetical esophageal gland cell secretory protein 12 | *Heterodera glycines* |
| *IKGN5RE01CUMWL_9* | $5 \times 10^{-11}$ | gb\|AAN52095.1 | putative esophageal gland cell secretory protein 31 | *Meloidogyne incognita* |
| *IKGN5RE01CYB5M_9* | $2 \times 10^{-38}$ | gb\|AAN08586.1 | putative esophageal gland cell secretory protein 20 | *Meloidogyne incognita* |
| *IKGN5RE01D01X1_3* | $2 \times 10^{-15}$ | gb\|AAQ10017.1 | putative esophageal gland cell secretory protein 3 | *Meloidogyne incognita* |

**Table S9.** *Cont.*

| Gene Name | *E* Value | ID | Function | Species |
|---|---|---|---|---|
| *IKGN5RE01D1FM5_5* | $6 \times 10^{-8}$ | gb\|AAN15809.1 | putative esophageal gland cell secretory protein 29 | *Meloidogyne incognita* |
| *IKGN5RE01D8493_3* | $1 \times 10^{-14}$ | gb\|AAQ10017.1 | putative esophageal gland cell secretory protein 3 | *Meloidogyne incognita* |
| *IKGN5RE01D9SN7_4* | $2 \times 10^{-9}$ | gb\|AAN15809.1 | putative esophageal gland cell secretory protein 29 | *Meloidogyne incognita* |
| *IKGN5RE01DC86S_1* | 0.0000004 | gb\|AAR37369.1 | putative esophageal gland cell secretory protein 38 | *Meloidogyne incognita* |
| *IKGN5RE01DHOKY_2* | $2 \times 10^{-31}$ | gb\|AAN15809.1 | putative esophageal gland cell secretory protein 29 | *Meloidogyne incognita* |
| *IKGN5RE01DT2MA_1* | $1 \times 10^{-12}$ | gb\|AAN15808.1 | putative esophageal gland cell secretory protein 28 | *Meloidogyne incognita* |
| *IKGN5RE01DV9U4_2* | $6 \times 10^{-31}$ | gb\|AAQ10016.1 | putative esophageal gland cell secretory protein 2 | *Meloidogyne incognita* |
| *IKGN5RE01DVQPH_5* | $5 \times 10^{-31}$ | gb\|AAF76925.1 | AF159590_1 hypothetical esophageal gland cell secretory protein 11 | *Heterodera glycines* |
| *IKGN5RE01DXDSG_8* | $4 \times 10^{-30}$ | gb\|AAQ10020.1 | putative esophageal gland cell secretory protein 6 | *Meloidogyne incognita* |
| *IKGN5RE01EDS56_3* | $2 \times 10^{-39}$ | gb\|AAF76925.1 | AF159590_1 hypothetical esophageal gland cell secretory protein 11 | *Heterodera glycines* |
| *IKGN5RE01EF5H1_2* | $7 \times 10^{-25}$ | gb\|AAQ10020.1 | putative esophageal gland cell secretory protein 6 | *Meloidogyne incognita* |
| *IKGN5RE01EFPLN_11* | $5 \times 10^{-9}$ | gb\|AAN15809.1 | putative esophageal gland cell secretory protein 29 | *Meloidogyne incognita* |
| *IKGN5RE01ELBUM_6* | $5 \times 10^{-11}$ | gb\|AAN15809.1 | putative esophageal gland cell secretory protein 29 | *Meloidogyne incognita* |
| *IKGN5RE01ENZFB_2* | $1 \times 10^{-29}$ | gb\|AAN08585.1 | putative esophageal gland cell secretory protein 19 | *Meloidogyne incognita* |
| *IKGN5RE01EQDR1_3* | $2 \times 10^{-16}$ | gb\|AAQ10017.1 | putative esophageal gland cell secretory protein 3 | *Meloidogyne incognita* |
| *IKGN5RE01ER4UR_8* | $5 \times 10^{-53}$ | gb\|AAF76926.1 | AF159591_1 hypothetical esophageal gland cell secretory protein 12 | *Heterodera glycines* |
| *IKGN5RE01EVNS1_9* | $7 \times 10^{-15}$ | gb\|AAN15809.1 | putative esophageal gland cell secretory protein 29 | *Meloidogyne incognita* |
| *IKGN5RE01EXQZU_3* | $3 \times 10^{-15}$ | gb\|AAQ10017.1 | putative esophageal gland cell secretory protein 3 | *Meloidogyne incognita* |
| *isotig01572* | $7 \times 10^{-19}$ | gb\|AAR37369.1 | putative esophageal gland cell secretory protein 38 | *Meloidogyne incognita* |
| *isotig05576* | $4 \times 10^{-76}$ | gb\|AAN15808.1 | putative esophageal gland cell secretory protein 28 | *Meloidogyne incognita* |
| *isotig05812* | $2 \times 10^{-20}$ | gb\|AAR37368.1 | putative esophageal gland cell secretory protein 37 | *Meloidogyne incognita* |
| *isotig07524* | 0 | gb\|AAN08587.1 | putative esophageal gland cell secretory protein 21 | *Meloidogyne incognita* |
| *isotig08163* | $1 \times 10^{-29}$ | gb\|AAQ10024.1 | putative esophageal gland cell secretory protein 9 | *Meloidogyne incognita* |
| *isotig08479* | $1 \times 10^{-103}$ | gb\|AAQ10018.1 | putative esophageal gland cell secretory protein 4 | *Meloidogyne incognita* |
| *isotig08832* | $5 \times 10^{-19}$ | gb\|AAR37369.1 | putative esophageal gland cell secretory protein 38 | *Meloidogyne incognita* |
| *isotig09791* | $3 \times 10^{-36}$ | gb\|AAN15806.1 | putative esophageal gland cell secretory protein 26 | *Meloidogyne incognita* |
| *isotig09833* | $2 \times 10^{-16}$ | gb\|AAR37369.1 | putative esophageal gland cell secretory protein 38 | *Meloidogyne incognita* |

**Table S9.** *Cont.*

| Gene Name | *E* Value | ID | Function | Species |
|---|---|---|---|---|
| *isotig10273* | $1 \times 10^{-53}$ | sp\|Q9BN19.1 | HSP6_HETGL RecName: full = putative esophageal gland cell secretory protein 6; flags: precursor gb | |
| *isotig10427* | $2 \times 10^{-44}$ | gb\|AAN08584.1 | putative esophageal gland cell secretory protein 18 | *Meloidogyne incognita* |
| *isotig10461* | $5 \times 10^{-42}$ | gb\|AAQ10018.1 | putative esophageal gland cell secretory protein 4 | *Meloidogyne incognita* |
| *isotig10924* | $2 \times 10^{-80}$ | gb\|AAQ10015.1 | putative esophageal gland cell secretory protein 1 | *Meloidogyne incognita* |
| *isotig11423* | $2 \times 10^{-43}$ | gb\|AAR37368.1 | putative esophageal gland cell secretory protein 37 | *Meloidogyne incognita* |
| *isotig11501* | $2 \times 10^{-52}$ | gb\|AAF76926.1 | AF159591_1 hypothetical esophageal gland cell secretory protein 12 | *Heterodera glycines* |
| *isotig11727.1* | $7 \times 10^{-60}$ | gb\|AAQ10020.1 | putative esophageal gland cell secretory protein 6 | *Meloidogyne incognita* |
| *isotig11727.2* | $7 \times 10^{-56}$ | gb\|AAN08579.1 | putative esophageal gland cell secretory protein 13 | *Meloidogyne incognita* |
| *isotig12059* | $2 \times 10^{-34}$ | gb\|AAN08586.1 | putative esophageal gland cell secretory protein 20 | *Meloidogyne incognita* |
| *IK2WLD402FRO9W_8* | $2 \times 10^{-22}$ | gb\|EJD74715.1 | hypothetical protein LOAG_18005 | *Loa loa* |
| *IK2WLD402G1OBP_3* | $1 \times 10^{-11}$ | ref\|XP_004888020.1 | predicted: transcriptional activator GLI3 isoform X2 | *Heterocephalus glaber* |
| *IK2WLD401D15KI_10* | $5 \times 10^{-38}$ | ref\|NP_498554.1 | protein NHR-10 | *Caenorhabditis elegans* |
